# WEE1 Dependency and Pejorative Prognostic Value in Triple‐Negative Breast Cancer

**DOI:** 10.1002/advs.202101030

**Published:** 2021-07-06

**Authors:** Alexandre de Nonneville, Pascal Finetti, Daniel Birnbaum, Emilie Mamessier, François Bertucci

**Affiliations:** ^1^ Laboratory of Predictive Oncology Institut Paoli‐Calmettes Comprehensive Cancer Center Aix‐Marseille Univ Marseille 13009 France

**Keywords:** cell cycle, expression, survival, triple‐negative breast cancer, WEE1

## Abstract

The WEE1 G2 checkpoint kinase acts as a negative cell cycle regulator for entry into mitosis (G2‐to‐M transition). This comment extends a recent Advanced Science paper by reporting higher WEE1‐dependency of triple negative breast cancer (TNBC) cell lines, pejorative prognostic value of *WEE1* expression in TNBC clinical samples as well as higher expression of biomarkers of sensitivity to WEE1 inhibitor.

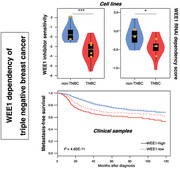

In their recent paper,^[^ ^1^ ^]^ Lamballe et al. identify the combination treatment with BCL‐XL and WEE1 inhibitors as a promising therapeutic approach in triple‐negative breast cancer (TNBC). TNBC is the most aggressive molecular subtype of breast cancer, but patients with TNBC have less benefited from recognized molecular targets than patients with other subtypes. In the adjuvant setting, the only systemic treatment currently approved remains chemotherapy.^[^ ^2^ ^]^ New systemic therapies are urgently needed. The Lamballe's study represents a promising new avenue for treatments targeting the cell cycle in TNBC. CDK4/6 inhibitors, which prevent phosphorylation of the RB tumor suppressor, thereby invoking cancer cell cycle arrest in G1, were recently approved for treatment of endocrine receptor‐positive (ER+) breast cancers. But TNBC has been considered a poor candidate because of frequent loss of RB expression or high cyclin E expression, both of which being expected to confer resistance to CDK4/6 inhibitors. Moreover, many TNBC cell lines showed resistance to CDK4/6 inhibition in vitro and in vivo.^[^ ^3^ ^]^ Recent works have pointed out that targeting other mitotic checkpoints in TNBC might help overcoming treatment resistance or synergizing drug effect.^[^ ^4^, ^5^ ^]^ The WEE1 G2 Checkpoint Kinase acts as a negative regulator of entry into mitosis (G2 to M transition) by protecting the nucleus from activated cyclin B1‐complexed CDK1, and is thought to exert protumorigenic functions by securing a tolerable level of genomic instability, an intrinsic feature of cancer cells.^[^ ^6^ ^]^ In order to reinforce and extend the Lamballe's results, we performed in silico analyses of WEE1 in large datasets of pre‐clinical and clinical breast cancer samples.

Using the genome‐wide CRISPR screen of 808 cell lines derived from many cancer types from the Broad Institute, we found that WEE1 was essential for the viability of almost all cell lines, independently from their lineage.^[^ ^7^, ^8^, ^9^ ^]^ Analysis was based on the CERES dependency score, a lower score indicating a higher likelihood that the gene is essential in a given cell line. In the subset of breast cancer cell lines, the CERES dependency scores for WEE1 were below the median of all pan‐essential genes (score ←1) in all cell lines, but no significant difference was observed between the TNBC versus non‐TNBC cell lines (data not shown). Such high‐level impact of WEE1 knock‐out reflects a very strong pan‐cancer cell WEE1‐dependency. We then investigated the consequence of WEE1 chemical inhibition, which is less drastic than the WEE1 knock‐out, in breast cancer cell lines according to the TN/non‐TN subtypes. For this, we used the primary PRISM Repurposing dataset containing the results of pooled‐cell line chemical‐perturbation viability screens for 4518 compounds tested against 578 cell lines.^[^ ^10^ ^]^ Consistently with the Lamballe's results, we observed an increased sensitivity to the MK‐1775 WEE1 inhibitor in the TNBC cell lines (*n* = 12, including 4 included in Lamballe's study) compared to the non‐TNBC cell lines (*n* = 10, including 2 included in Lamballe's study) (*P* = 6.00E‐04, Mann‐Whitney test; **Figure** **1**A; Table S1, Supporting Information).

**Figure 1:**
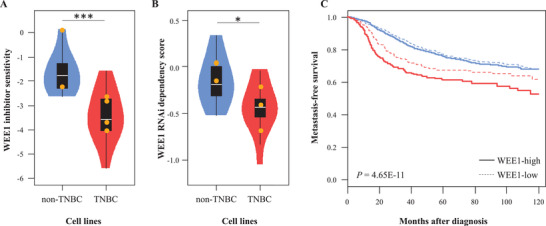
WEE1‐dependence in TNBC: A) Box plot of WEE1 inhibitor MK‐1775 sensitivity (logfold change values relative to DMSO) in breast cancer cell lines: TNBC (*n* = 12) versus non‐TNBC (*n* = 10). The statistical significance was assessed using the Mann‐Whitney test. The orange dots represent the cell lines included in the Lamballe's study. B) Box plot of RNAi DEMETER2 dependency score in breast cancer cell lines: TNBC (*n* = 12) versus non‐TNBC (*n* = 10). The statistical significance was assessed using the Mann‐Whitney test. The orange dots represent the cell lines included in the Lamballe's study. C/ Kaplan‐Meier metastasis‐free survival (MFS) in patients with breast cancer (*n* = 3454) according to the WEE1 expression‐based class in TNBC (red curves) and in non‐TNBC (blue curves). The statistical significance was assessed using the log‐rank test. *, *P* < 0.05; ***, *P* < 0.001.

Both CRISPR and MK‐1775 WEE1 inhibitor analyses suggest that a proper balance of WEE1 inhibition is necessary to achieve cancer‐specific lethality in TNBC cell lines. To support this hypothesis, we used the DEMETER2 algorithm, applied to three large‐scale RNA interference (RNAi) screening datasets Marcotte et al.: the Broad Institute Project Achilles, the DRIVE Novartis Project, and the breast cell line dataset.^[^ ^11^ ^]^ RNAi dependency analysis in the 22 above‐analyzed breast cancer cell lines revealed very comparable results to the MK‐1775 WEE1 inhibitor assay, with, again, a higher WEE1 dependency in the TNBC cell lines compared to the non‐TNBC cell lines (*P* = 3.58E‐02, Mann‐Whitney test; Figure 1B: Table S1, Supporting Information).

The clinical relevance of WEE1 expression in breast cancer has been little studied and to our knowledge has never been assessed in large series of TNBC. We thus retrospectively examined the normalized *WEE1* mRNA expression in 8636 primary breast cancers, including 1847 TNBC, gathered from 36 public gene expression data sets.^[^ ^12^ ^]^ In TNBC, high *WEE1* expression (defined as expression above median expression level in the whole data set) was associated with high pathological grade, pT2 size, and basal‐like 1 and mesenchymal Lehmann subtypes^[^ ^13^ ^]^ (Table S2, Supporting Information). A total of 692 TNBC patients were informative for metastasis‐free survival (MFS). The 5‐year MFS was 68% (95%CI 62–74) in the “WEE1‐low” class versus 61% (95%CI 56‐67) in the “WEE1‐high” class (*P* = 3.64E‐02, log‐rank test; Figure 1C). In univariate analysis, the hazard ratio (HR) for metastatic relapse was 1.35 (95%CI 1.02‐1.78) in the “WEE1‐high” class versus the “WEE1‐low” class (*P* = 3.72E‐02, Wald test). In multivariate analysis, *WEE1* expression remained associated with MFS (HR 1.37, 95%CI (1.03‐1.81); *P* = 2.92E‐02, Wald test), suggesting independent prognostic value (**Table** **1**).

**Table 1:** Univariate and multivariate Cox regression analysis for MFS in TNBC (HR, hazards ratio)

		Univariate			Multivariate		
		*n*	HR [95%CI]	*P*‐value^a)^	*n*	HR [95%CI]	*P*‐value^a)^
Patients' age	>50 vs< = 50	534	1.22 [0.84–1.78]	0.291			
Pathological grade	2 vs 1	343	4.69 [0.63–34.95]	0.117			
	3 vs 1		6.16 [0.86–44.31]				
Pathological axillary lymph node status	positive vs negative	524	1.21 [0.83–1.77]	0.314			
Pathological tumor size	pT2 vs pT1	475	1.13 [0.72–1.76]	0.108			
	pT3 vs pT1		1.99 [1.03–3.83]				
Pathological tumor type	ILC vs IDC	343	2.13 [0.52–8.84]	0.215			
	Other vs IDC		0.53 [0.21–1.34]				
Lehmann TN BC subtype	Basal‐like 2 vs basal‐like 1	692	1.45 [0.94–2.23]	0.095	692	1.53 [0.99‐2.38]	0.055
	Mesenchymal vs basal‐like 1	692	1.78 [1.23–2.57]	**2.20E‐03**	692	1.79 [1.24–2.58]	**1.99E‐03**
	Luminal AR vs basal‐like 1	692	1.30 [0.89–1.91]	0.179	692	1.34 [0.91–1.98]	0.133
*WEE1* mRNA class	High vs low	692	1.35 [1.02–1.78]	**3.72E‐02**	692	1.37 [1.03–1.81]	**2.92E‐02**

^a)^ Wald test.

Given the differential sensitivity of TNBC versus non‐TNBC cell lines to WEE1 modulation, we assessed the prognostic value of *WEE1* mRNA expression in the 2762 non‐TNBC patients of our dataset informative for MFS. No MFS difference was observed between the “WEE1‐high” and the “WEE1‐low” classes (*P* = 0.816, log‐rank test; Figure 1C). The Cox interaction test for MFS between *WEE1* expression and TNBC versus non‐TNBC subtypes was significant (*P* = 7.17E‐03, Wald test). Finally, the TNBC “WEE1‐high” samples displayed, as compared to the “WEE1‐low” samples, higher *CCNE1* expression (*p* = 4.00E‐08, Mann‐Whitney test) and more frequent chromosomal instability (assessed by the Carter's gene expression signature; *P* = 3.86E‐16, Fisher's exact test), two markers recently associated with higher sensitivity to MK‐1775 inhibitor in breast cancer models.^[^ ^14^ ^]^

Thus, our data not only confirm the increased sensitivity of TNBC cell lines to the MK‐1775 WEE1 inhibitor on a larger panel of cell lines, but also show the higher WEE1‐dependency of TNBC cell lines and the independent pejorative prognostic value of *WEE1* expression in a large series of TNBC clinical samples. Altogether, these results nicely complement the Lamballe's results and further support the development of WEE1‐targeting therapies in TNBC.

## Conflict of Interest

The authors declare no conflict of interest.

## Supporting information

Supporting Information

Supporting Information

## Data Availability

The data that support the findings of this study are openly available at the CCLE portal (www.broadinstitute.org/ccle) and DepMap portal (http://www.depmap.org). genome‐wide CRISPR Avana 21Q1 public dataset (gene effects, CERES scores) was downloaded from https://figshare.com/articles/dataset/public_21q1/13681534. PRISM Repurposing 19Q3 Primary Screen was downloaded from https://ndownloader.figshare.com/files/17741420. DEMETER2 Data v6 was downloaded from https://ndownloader.figshare.com/files/13515395.

## References

[1] F. Lamballe, F. Ahmad, Y. Vinik, O. Castellanet, F. Daian, A. Müller, U. A. Köhler, A. Bailly, E. Josselin, R. Castellano, C. Cayrou, E. Charafe‐Jauffret, G. B. Mills, V. Géli, J. Borg, S. Lev, F. Maina, Adv. Sci. 2021, 8, 2003049. 10.1002/advs.202003049 PMC7856896 33552868

[2] A. de Nonneville, P. Finetti, J. Adelaide, É. Lambaudie, P. Viens, A. Gonçalves, D. Birnbaum, E. Mamessier, F. Bertucci, Cancers 2019, 11, 1158. 10.3390/cancers11081158 PMC6721506 31412533

[3] S. Pernas, S. M. Tolaney, E. P. Winer, S. Goel, Ther. Adv. Med. Oncol. 2018, 10, 175883591878645. 10.1177/1758835918786451 PMC6050811 30038670

[4] S. Moens, P. Zhao, M. F. Baietti, O. Marinelli, D. Van Haver, F. Impens, G. Floris, E. Marangoni, P. Neven, D. Annibali, A. A. Sablina, F. Amant, Sci. Rep. 2021, 11, 3176. 33542435 10.1038/s41598-021-82780-6 PMC7862668

[5] J. C. Liu, L. Granieri, M. Shrestha, D.‐Y. Wang, I. Vorobieva, E. A. Rubie, R. Jones, Y. Ju, G. Pellecchia, Z. Jiang, C. A. Palmerini, Y. Ben‐David, S. E. Egan, J. R. Woodgett, G. D. Bader, A. Datti, E. Zacksenhaus, Cell Rep. 2018, 23, 112. 29617654 10.1016/j.celrep.2018.03.039 PMC9357459

[6] A. Ghelli Luserna di Rorà, C. Cerchione, G. Martinelli, G. Simonetti, J. Hematol. Oncol. 2020, 13, 126. 32958072 10.1186/s13045-020-00959-2 PMC7507691

[7] J. M. Dempster, J. Rossen, M. Kazachkova, J. Pan, G. Kugener, D. E. Root, A. Tsherniak, bioRxiv 2019, 720243, 10.1101/720243.

[8] R. M. Meyers, J. G. Bryan, J. M. McFarland, B. A. Weir, A. E. Sizemore, H. Xu, N. V. Dharia, P. G. Montgomery, G. S. Cowley, S. Pantel, A. Goodale, Y. Lee, L. D. Ali, G. Jiang, R. Lubonja, W. F. Harrington, M. Strickland, T. Wu, D. C. Hawes, V. A. Zhivich, M. R. Wyatt, Z. Kalani, J. J. Chang, M. Okamoto, K. Stegmaier, T. R. Golub, J. S. Boehm, F. Vazquez, D. E. Root, W. C. Hahn, A. Tsherniak, Nat. Genet. 2017, 49, 1779. 29083409 10.1038/ng.3984 PMC5709193

[9] Broad DepMap. Public_21q1, 2021, 12817867672 Bytes, 10.6084/M9.FIGSHARE.13681534.V1. Accessed 18 February 2021.

[10] S. M. Corsello, R. T. Nagari, R. D. Spangler, J. Rossen, M. Kocak, J. G. Bryan, R. Humeidi, D. Peck, X. Wu, A. A. Tang, V. M. Wang, S. A. Bender, E. Lemire, R. Narayan, P. Montgomery, U. Ben‐David, Y. Chen, M. G. Rees, N. J. Lyons, J. M. McFarland, B. T. Wong, L. Wang, N. Dumont, P. J. O'Hearn, E. Stefan, J. G. Doench, H. Greulich, M. Meyerson, F. Vazquez, A. Subramanian, J. A. Roth, J. A. Bittker, J. S. Boehm, C. C. Mader, A. Tsherniak, T. R. Golub, bioRxiv 2019, 730119. 10.1101/730119.

[11] J. M. McFarland, Z. V. Ho, G. Kugener, J. M. Dempster, P. G. Montgomery, J. G. Bryan, J. M. Krill‐Burger, T. M. Green, F. Vazquez, J. S. Boehm, T. R. Golub, W. C. Hahn, D. E. Root, A. Tsherniak, Nat. Commun. 2018, 9, 4610. 30389920 10.1038/s41467-018-06916-5 PMC6214982

[12] F. Bertucci, P. Finetti, A. Goncalves, D. Birnbaum, npj Breast Cancer 2020, 6, 8. 32195331 10.1038/s41523-020-0151-5 PMC7060267

[13] B. D. Lehmann, J. A. Bauer, X. Chen, M. E. Sanders, A. B. Chakravarthy, Y. Shyr, J. A. Pietenpol, J. Clin. Invest. 2011, 121, 2750. 21633166 10.1172/JCI45014 PMC3127435

[14] X. Chen, K.‐H. Low, A. Alexander, Y. Jiang, C. Karakas, K. R. Hess, J. P. W. Carey, T. N. Bui, S. Vijayaraghavan, K. W. Evans, M. Yi, D. C. Ellis, K.‐L. Cheung, I. O. Ellis, S. Fu, F. Meric‐Bernstam, K. K. Hunt, K. Keyomarsi, Clin. Cancer Res. 2018, 24, 6594. 30181387 10.1158/1078-0432.CCR-18-1446 PMC6317865

