# Supplementary material for: WEE1 Dependency and Pejorative Prognostic Value in Triple‐Negative Breast Cancer
Source: Adv Sci (Weinh). 2021 Jul 6;8(17):2101030. doi: 10.1002/advs.202101030 (PMC8425927; doi:10.1002/advs.202101030)
Supplement: Supplementary file 2 — Supporting Information [file ADVS-8-2101030-s002.pdf]

© 2021 Wiley-VCH GmbH

ADVANCED SCIENCE
Open Access

Supporting Information

for *Adv. Sci.*, DOI: 10.1002/advs.202101030

# WEE1 Dependency and Pejorative Prognostic Value in Triple-Negative Breast Cancer

*Alexandre De Nonneville, Pascal Finetti, Daniel Birnbaum, Emilie Mamessier, and François Bertucci**
